# Supplementary material for: Can Modern Molecular Modeling Methods Help Find the Area of Potential Vulnerability of Flaviviruses?
Source: Int J Mol Sci. 2022 Jul 13;23(14):7721. doi: 10.3390/ijms23147721 (PMC9316223; doi:10.3390/ijms23147721)
Supplement: Supplementary file 1 [file ijms-23-07721-s001.zip › ijms-1790218-supplementary.pdf]

Supplementary materials:

```
                             D I                                         D II
                                      10        20        30        40        50        60        70        80
                             ....|....|....|....|....|....|....|....|....|....|....|....|....|....|....|....|
Tick-borne encephalitis virus SRCTHLENRDFVTGTQGTTRVTLVLELGGCVTITAEGKPSMDVWLDAIYQENPAKTREYCLHAKLSDTKVAARCPTMGPA
Japanese encephalitis virus   FN.LGMG...IE.AS.A.W.D....GDS.L..M.ND..TL..RMIN.EASQL.EV.S..Y..SVT.ISTV.....T.E.
West Nile virus               FN.LGMS...LE.VS.A.W.D....GDS....MSKD..TI..KMMNMEAA.L.DV.S..YL.SV..LSTR.A.....E.
Yellow fever virus            AH.IGITD..IE.VH.G.W.SAT..QDK...VM.PD...L.IS.ETVAIDR..EV.KV.YN.V.THV.INDK..ST.E.
Zika virus                    I..IGVS....E.MS.G.W.DV...H.....VM.QD..TV.IE.VTTTVS.M.EV.S..YE.SI..MASDS....Q.E.
Dengue virus 1                M..VGIG....E.LS.A.W.DV...H.S...TM.KD..TL.IE.LKTEVT...VL.KL.IE..I.N.TTDS....Q.E.
Dengue virus 2                M..IGMS....E.VS.GSW.DI...H.S...TM.KN..TL.FE.IKTEAKQ..TL.K..IE...TN.TTES....Q.EP
Dengue virus 3                M..VGVG....E.LS.A.W.DV...H.....TM.KN..TL.IE.QKTEATQL.TL.KL.IEG.ITNITTDS....Q.E.
Dengue virus 4                M..VGVG....E.VS.GAW.D....H.....TM.Q...TL.FE.TKTTAKEV.LL.T..IE.SI.NITT.T....Q.EP

                             D II            FL          D II                    D I
                                      90       100       110       120       130       140       150       160
                             ....|....|....|....|....|....|....|....|....|....|....|....|....|....|....|....|
Tick-borne encephalitis virus TLAEEHQGGTVCKRDQSDRGWGNHCGLFGKGSIVACVKAACEAKKKATGHVYDANKIVYTVKVEPHTGDYVAANE-----
Japanese encephalitis virus   HNEKRADSSY...QGFT.....G.........DT.A.FS.TS.--.I.RTIQPEN.K.E.GIFV.GTTTSENHGNYSAQ
West Nile virus               HNEKRADPAF...QGVV.....G.........DT.A.F..TT.--...WIIQKEN.K.E.AIFV.GPTT.ESHG----K
Yellow fever virus            H....NE.DNA...TY......G............A.FT.AKS--MSLFEV.QT..Q.VIRAQL.V.AKQENWN-----
Zika virus                    Y.DKQSDTQY....TLV.....G........L.T.A.FT.SK.--M..KSIQPENLE.RIMLSV.GSQHSGMIG----Y
Dengue virus 1                ..V..QDTNF..R.TFV.....G........LIT.A.FK.VT.--LE.KIVQYENLK.S.I.TV....QH-QVG----N
Dengue virus 2                S.N..QDKRF...HSMV.....G.......G..T.AMFR.KKN--ME.K.VQPENLE..IVIT..S.EEH-.VG----N
Dengue virus 3                V.P..QDQNY...HTYV.....G........L.T.A.FQ.LEP--IE.K.VQYENLK...IITV....QH-QVG----N
Dengue virus 4                Y.K..QDQQYI.R..VV.....G.......GV.T.A.FS.SG.--I..NLVQIENLE...V.TV.N..TH-.VG----N

                             D I                         D II
                                     170       180       190       200       210       220       230       240
                             ....|....|....|....|....|....|....|....|....|....|....|....|....|....|....|....|
Tick-borne encephalitis virus -THSGRKTASFTVSSEKTILTMGEYGDVSLLCRVASGVDLAQTVILELDKTVEHLPTAWQVHRDWFNDLALPWKHEGAQ-
Japanese encephalitis virus   VGA.QAAKFTI.PNAPSIT.KL.D..E.T.D.EPR..LKTEAFYVMTVGS------KSFL...E..H......TSPSST-
West Nile virus               IGATQAGRF.I.P.APSYT.KL....E.TVD.EPR..I.TSAYYVMSVGE------KSFL...E..M..N...SSA.ST-
Yellow fever virus            ---TDI..LK.DAL.GSQEVEFIG..KAT.E.Q.QTA..FGNSY.A.MET------ES.I.D.Q.AQ..T...QSGSGG-
Zika virus                    E.DED.AKVEV.PN.PRAEA.L.GF.SLG.D.EPRT.L.FSDLYY.TMNN------KH.L..KE..H.IP...HAGADTG
Dengue virus 1                E.TEHGT..TI.PQAPTSEIQLTD..ALT.D.SPRT.L.FNEM.L.TMK.------KS.L..KQ..L..P...TSGASTS
Dengue virus 2                D.GKHG.EIKI.PQ.SI.EAELTG..T.TME.SPRT.L.FNEM.L.QMEN------K..L...Q..L..P...LPGADTQ
Dengue virus 3                E.--QGV..EI.PQAST.EAILP...TLG.E.SPRT.L.FNEMIL.TMKN------K..M...Q..F..P...ASGATTE
Dengue virus 4                D.SNHGV..MI.PR.PSVEVKLPD..ELT.D.EPR..I.FNEMILMKMK.------KT.L..KQ..L..P...TAGADTS
```

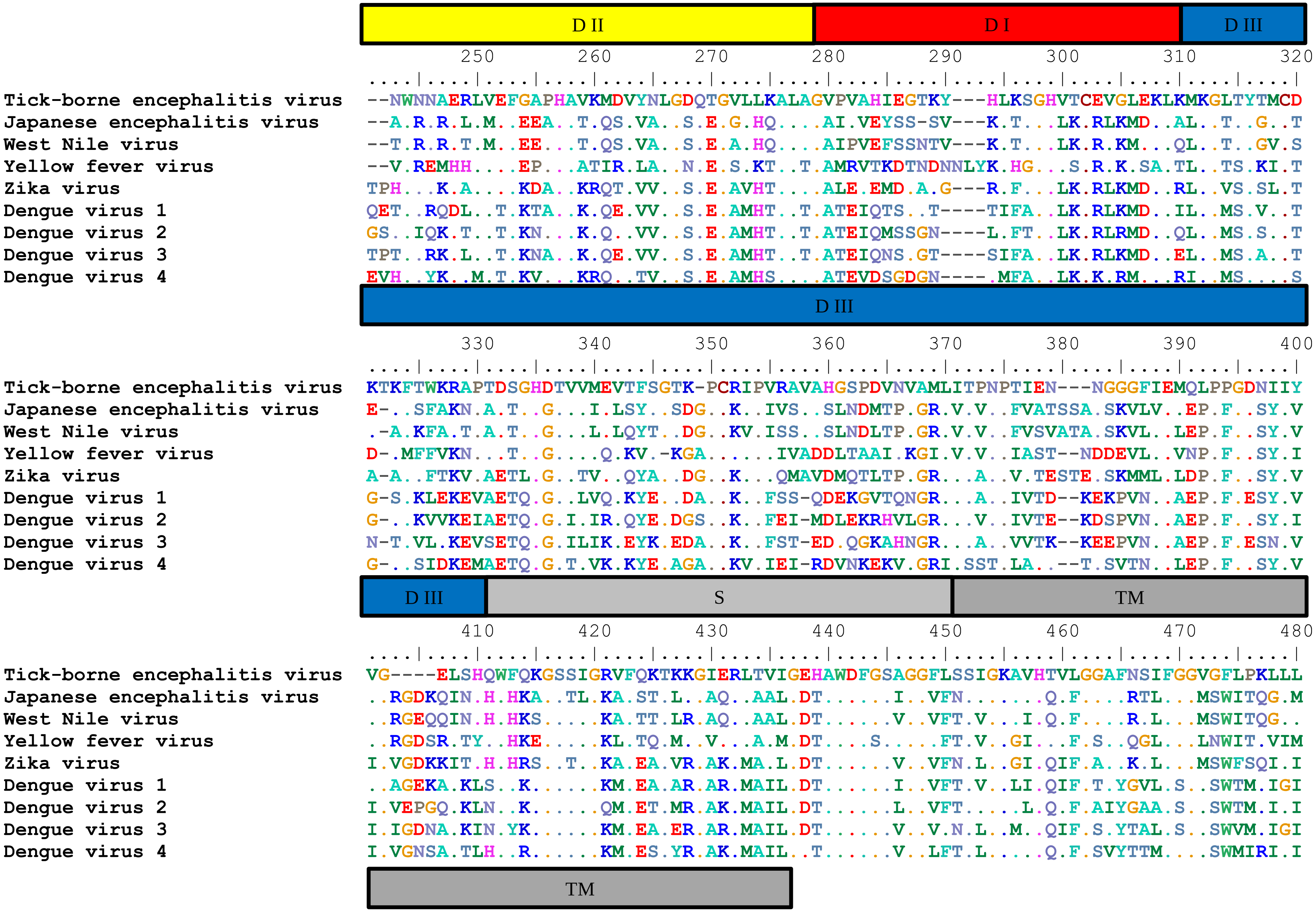
D II
D I
D III
250 260 270 280 290 300 310 320
Tick-borne encephalitis virus
Japanese encephalitis virus
West Nile virus
Yellow fever virus
Zika virus
Dengue virus 1
Dengue virus 2
Dengue virus 3
Dengue virus 4
D III
330 340 350 360 370 380 390 400
D III
S
TM
410 420 430 440 450 460 470 480
TM

```
                                          490       500       510
                               ....|....|....|....|....|....|....|.
Tick-borne encephalitis virus  GVALAWLGLNMRNPTMSMSFLLAGGLVLAMTLGVGA
Japanese encephalitis virus    .AL.L.M.V.A.DRSIALA..AT..VLVFLATN.H.
West Nile virus                .AL.L.M.I.A.DRSIA.T..AV..VL.FLSVN.H.
Yellow fever virus             .AV.I.V.I.T..M.....MI.V.VIMMFLS.....
Zika virus                     .TL.V.....TK.GSI.LTC.AL..VMIFLSTA.S.
Dengue virus 1                 .IL.T.....S.STSL..TCIAV.MVT.YLGVM.Q.
Dengue virus 2                 ..IIT.I.M.S.STSL.VTLV.V.IVT.YLGVM.Q.
Dengue virus 3                 ..L.T.I...SK.TS..F.CIAI.IIT.YLGAV.Q.
Dengue virus 4                 .FLVL.I.T.S..TS.A.TCIAV..IT.FLGFT.Q.
```

Figure S1 Alignment of amino acid sequences of surface protein E TBEV, JEV, WNV, YF, ZIKV and DENV (I-IV)

Supplementary materials:

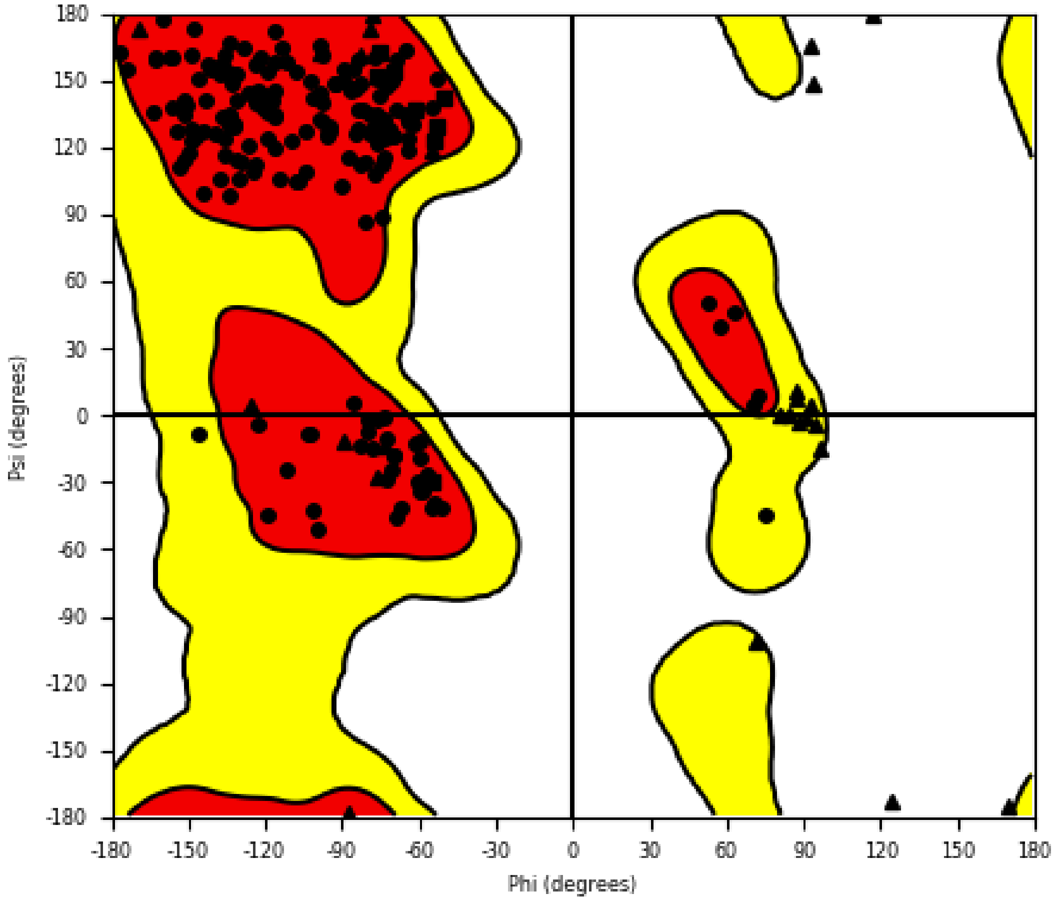

Figure S2 Ramachandran plot

The Root Mean Square Deviation (RMSD is used to measure the average change in displacement of a selection of atoms for a particular frame with respect to a reference frame. It is calculated for all frames in the trajectory. The RMSD for frame x is:

$$RMSD_x = \sqrt{\frac{1}{N}\sum_{i=1}^{N}\left(r_i'(t_x) - r_i(t_{ref})\right)^2}$$

where $\boldsymbol{N}$ is the number of atoms in the atom selection; $\boldsymbol{t_{ref}}$ is the reference time (in our case $\boldsymbol{t_{ref}}$ = 100 ns), (typically the first frame is used as the reference and it is regarded as time t = 0); and $\boldsymbol{r'}$ is the position of the selected atoms in frame $\boldsymbol{x}$ after superimposing on the reference frame, where frame $\boldsymbol{x}$ is recorded at time $\boldsymbol{t_x}$. The procedure is repeated for every frame in the simulation trajectory.

Changes of the order of 1-3 Å are perfectly acceptable for small proteins. Changes much larger than that, however, indicate that the protein is undergoing a large conformational change during the simulation.

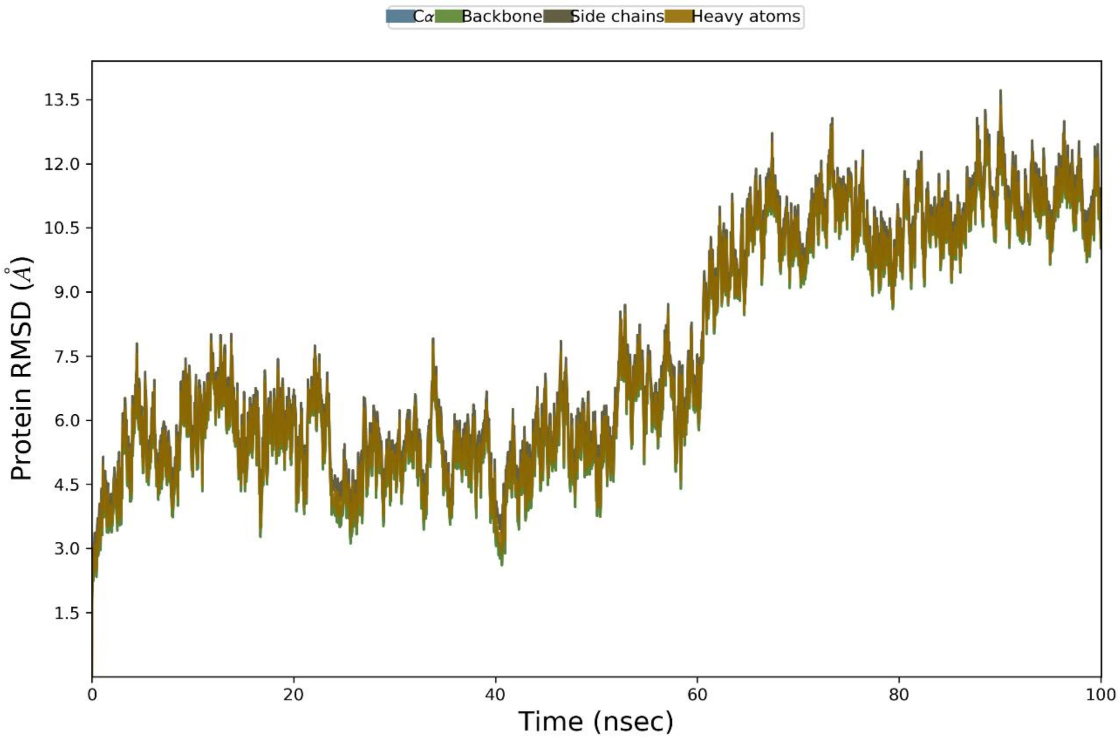

Figure S3 RMSD value for T-10H10 complex

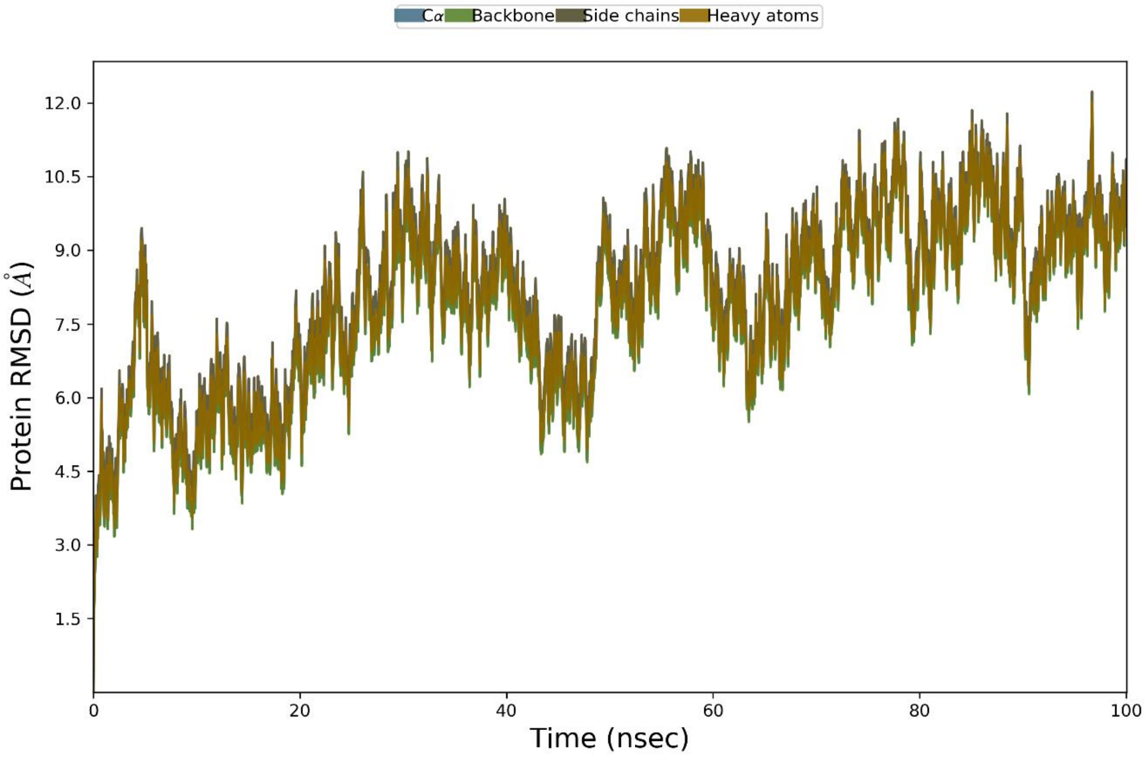

Figure S4 RMSD value for Z-10H10 complex

Supplementary materials:

Table S1 Primers used to make recombinant plasmids

Name	Sequence
TBEV-F	AAAAAACCATGGACTCACGATGCACACATCTGGAAAA
TBEV-R	AAAAACTCGAGTCGGCACAACAAGGACACGT
DENV-F	AAAAAAGGATCCATGCGTTGTGTTGGTGTTGGCAAT
DENV-R	AAAAAACTCGAGGGCGCTACATTCCAGACCTAAGGTGC
WNV-F	AAAAAAGGATCCTTCAACTGCCTTGGAATGAGCA
WNV-R	AAAAAAACTCGAGCGTCACTTCTCCATATTCTCCAAGC
ZIKV-F	AAAAAACCATGGATCAGGTGCATTGGAGTCAGCAATAGAG
ZIKV-R	AAAAACTCGAGTTCACAGTCAAGTCCTAAGCTTCCA
AbHC_MH1_F	SARGTNMAGCTGSAGSAGTC
AbLC_5XMk_F	GAYATTGTGMTSACMCARWCTMCA
AbHC_mIgG2B_R	AGGGGCCAGTGGATAGACTGATGG
AbLC_3XKc_R	GGATACAGTTGGTGCAGCATC

Supplementary materials:

Table S2 Amino acid sequences of the light and heavy chains of the 10H10 antibody

Name	Sequence
VH	VKLEESGGGLVKPGGSLKLSCAASGFSFSDYYMYWVRQTPEKRLEWVATI<br>SDGGSHTSYRDSVKGRFTISRDNGKNNLYLQMSSLKSEDTAMYYCVRGA<br>YWGQGTLVTVSSA
VL	DIVLTQTPLTLSVTIGQPASISCKSSQSLLDSDGKTYLNWLLQRPGQSPKRLI<br>YLVSKLDSGVPDRFTGGGSGTDFTLKISRVEAEDLGVYYCWQGTHFPQTF<br>GGGTKLEIK

Supplementary materials:

Table S3 Molecular docking result specific interaction between amino acid of the FL of TBEV and ZIKV and 10H10: chain A is amino acid sequence of the FL of TBEV and ZIKV; chain B and L correspond to 10H10 antybody; HB – hydrogen bond; SB – salt bridge; pi-pi – stacking interaction.

TBEV F	10H10				ZIKV F	10H10			
Chain A	Chain H	Specific Interactions	Chain L	Specific Interactions	Chain A	Chain H	Specific Interactions	Chain L	Specific Interactions
Docking position (start position)									
T76	×	×	K36	HB	S72,	×	×	K36	HB
D98	Y38	HB	×	×	D98	×	×	K36	SB
					R99	Y40	HB	Q116	HB
G102	Y40	HB	×	×					
H104	×	×	F114	Pi-Pi	G104	×	×	R52	HB
H104	×	×	D31	HB	C105	×	×	R52	HB
					F108	Y40	Pi-Pi	×	×
					K110	D36<br>D58	HB<br>HB, SB	×	×
					K251	×	×	D31<br>D34<br>Y38	HB, SB<br>SB<br>HB
Position corresponds to 40000 ps									
R73	D58	HB	×	×	S72	×	×	D34	HB
C74	D58	HB	×	×	D98	×	×	K36	HB, SB
T76	Y38	HB	×	×	G100	Y40	HB	×	×
R99	D36	HB	×	×	W101	R106	HB	W105	Pi-Pi
W101	×	×	W105	Pi-Pi	G104	×	×	N40	HB
H104	Y37	Pi-Pi	×	×	C105	×	×	R52	HB
H104	Y38	HB	×	×	G106	A116	HB	×	×
G106	×	×	Q116	HB	K251	×	×	D31<br>D34	HB<br>SB
Position corresponds to 100000 ps									
A72	Y37	HB	×	×	S72	×	×	K36	HB
R73	D36	HB, SB	×	×	T76	×	×	Y55	HB
D98	×	×	K36	HB, SB	D98	×	×	K36	HB, SB
R99	Y37	HB	×	×	G100	Y40	HB	×	×
H104	Y38	HB	×	×	W101	R106	HB	×	×
H104	Y40	Pi-Pi	×	×	G104	×	×	N40<br>L56	HB
					C105	×	×	R52	HB
					G106	A116	HB	×	×
					K251	×	×	D31<br>D36	HB, SB
